# Chrysin Protects Rat Kidney from Paracetamol-Induced Oxidative Stress, Inflammation, Apoptosis, and Autophagy: A Multi-Biomarker Approach

**DOI:** 10.3390/scipharm85010004

**Published:** 2017-01-25

**Authors:** Fatih Mehmet Kandemir, Sefa Kucukler, Eyup Eldutar, Cuneyt Caglayan, İlhami Gülçin

**Affiliations:** 1 Department of Biochemistry, Faculty of Veterinary Medicine, Ataturk University, 25240 Erzurum, Turkey; fmehmet.kandemir@atauni.edu.tr (F.M.K.); sefa_kcklr@hotmail.com (S.K.); eyup.eldutar@arilab.com.tr (E.E.); 2 Department of Biochemistry, Faculty of Veterinary Medicine, Bingol University, 12000 Bingol, Turkey; ccaglayan@bingol.edu.tr; 3 Department of Chemistry, Faculty of Sciences, Ataturk University, 25240 Erzurum, Turkey

**Keywords:** chrysin, paracetamol, nephrotoxicity, oxidative stress, inflammation

## Abstract

Paracetamol (PC) is a safe analgesic and antipyretic drug at therapeutic doses, and it is widely used in clinics. However, at high doses, it can induce hepatotoxicity and nephrotoxicity. Chrysin (CR) is a natural flavonoid that has biological activities that include being an antioxidant, an anti-inflammatory, and an anti-cancer agent. The main objective of this study was to investigate the efficacy of CR against PC-induced nephrotoxicity in rats. CR was given orally via feeding needle to male Sprague Dawley rats as a single daily dose of 25 or 50 mg/kg for six days. PC was administered orally via feeding needle as a single dose on the sixth day. PC caused significant glutathione depletion, lipid peroxidation, increased serum toxicity markers (serum urea and creatinine), and reductions in activities of antioxidant enzymes (superoxide dismutase — SOD, catalase — CAT, and glutathione peroxidase — GPx). The renal protective effect of CR was associated with decreasing the regulation of serum renal toxicity markers and increasing the regulation of antioxidant enzyme activities. Additionally, PC led to significant increases in the levels of inflammatory markers including tumour necrosis factor-alpha (TNF-α), interleukin-1β (IL-1β) and interleukin-33 (IL-33). Furthermore, PC induced apoptotic tissue damage by increasing cysteine aspartate-specific protease-3 (caspase-3) activity and autophagic tissue damage by increasing the expression of light chain 3B (LC3B). CR therapy significantly decreased these values in rats. This study demonstrated that CR has antioxidant, anti-apoptotic, anti-inflammatory and anti-autophagic effects on PC-induced kidney toxicity in rats.

## 1. Introduction

Paracetamol (PC) is a commonly used as analgesic and antipyretic drug [1]. It is safe at therapeutic levels, but at high doses it can lead to undesirable side effects such as hepatotoxicity and nephrotoxicity [2]. Although high doses of PC establish a conjugated bond with glucuronic acid or sulphate, a significant portion is metabolized by the cytochrome P450 system. This probably leads to the production of reactive toxic metabolites such as *N*-acetyl-*p*-benzoquinone imine (NAPQI) interacting with sulfhydryl groups in the glutathione (GSH) molecule [3,4]. Therefore, PC induces a depletion of cellular GSH stores. Binding cellular proteins, the remaining portion of NAPQI begins lipid peroxidation and ultimately induces kidney damage [5,6]. Therefore, PC toxicity is determined by the amount of NAPQI produced and the inadequate GSH for PC detoxification [7]. Autophagia is a eukaryotic process that maintains cellular homeostasis by using protein aggregates and dead organelles in case cells are deprived of nutrients [8]. In the kidney and other organs, it is observed that autophagy is a critical pathway for both eliminating damaged mitochondria and promoting cellular repair after cell damage [9]. Decreases in antioxidant enzyme levels and increases in the production of reactive oxygen species (ROS) are observed as a result of PC-induced tissue damage [10]. Oxidative stress and the formation of free radicals in PC-induced kidney and liver damage have been reported in several studies [4,6]. Furthermore, some studies have reported that high doses of PC reduce tubular epithelial cell vitality by leading to kidney toxicity [11,12]. Hence, one of the alternative therapeutic approaches against PC-induced kidney toxicity is the use of natural compounds that are used for medical purposes and have antioxidant activities [13].

The flavonoids that play a role as antioxidants in biological systems are natural phenolic compounds present in fruit and vegetable species [14]. Flavonoids have many useful effects that include being antioxidants and having anti-bacterial, anti-cancer, anti-mutagenic and anti-inflammatory properties [15,16,17].

Chrysin (5,7-dihydroxyflavone; CR) is a natural flavonoid available in many plant extracts including the blue passion (*Passiflora caerulea*) flower, bee propolis and honey [18]. It has free radical elimination effects with the hydroxyl groups of CR in the fifth and the eighth positions [19]. Like the flavonoids, CR has several pharmacological effects such as being an antioxidant, and having anti-inflammatory, anti-aging, anti-cancer and anti-hypertensive properties [20]. Additionally, it has been reported that CR protects the liver from chemotherapeutic drugs and other agents that induce toxicity [21]. The aim of this study was to investigate the antioxidant, anti-apoptotic, anti-inflammatory and anti-autophagic effects of CR against PC-induced nephrotoxicity in rats.

## 2. Materials and Methods

### 2.1. Drugs and Chemicals

Parol^®^ (500 mg/tablet), the source of PC was purchased from Atabay (Atabay Chemical Industry, Istanbul, Turkey). It was suspended in distilled water. CR was purchased from Sigma-Aldrich Chemical Company, (St. Louis, MO, USA). CR was suspended in distilled water. All other chemicals used in the study were purchased from the Sigma-Aldrich Chemical Company. Administration of PC 500 mg/kg was as a single dose orally [22]. In this study, CR 25 mg/kg and CR 50 mg/kg were prepared according to previous studies [23].

### 2.2. Animals

In this study, 35 adult male Sprague-Dawley rats, weighing about 250–300 g were provided from the Animal Laboratory at the Experimental Research Centre of Ataturk University, Erzurum, Turkey. The rats were given free access to a standard pellet diet and tap water ad libitum and were acclimated to the laboratory conditions for one week before starting the experiment [24]. They were maintained in standard housing facilities (temperature: 24 ± 1°C, relative humidity range: 45% ± 5%, and a 12 h light/12 h dark cycle). The study protocol was approved by the Local Animal Care Committee of Ataturk University, Erzurum, Turkey (Protocol No: 2016-2/52).

### 2.3. Experimental Design

All of the rats used in this experiment were randomly divided into five groups of seven rats each. The five groups are described below.

Control group: the rats were given an oral physiological saline solution for 6 days.

CR group: the rats were orally given CR (50 mg/kg) for 6 days.

PC group: Physiological saline solution was administered orally for 6 days. On the 6th day, a single dose of PC (500 mg/kg) was administered to induce kidney toxicity.

PC+CR-25 group: CR (25 mg/kg) was administered orally for 6 days. On the 6th day, a single dose of PC (500 mg/kg) was administered 30 min after the CR application.

PC+CR-50 group: CR (50 mg/kg) was administered orally for 6 days. On the 6th day, a single dose of PC (500 mg/kg) was administered 30 min after the CR application.

All rats were sacrificed 24 h following their final respective administration. Rats were sacrificed by decapitation under sevoflurane (Sevorane liquid 100%, Abbott Laboratory, Istanbul, Turkey) anesthesia.

### 2.4. Sample Collection

Blood samples were transferred into clean dry test tubes without anticoagulant and allowed to clot at room temperature for 10 min. Afterwards the blood was centrifuged at 1200× *g* for 10 min at 4°C and serum was obtained for analysis of urea and creatinine concentrations for biochemical analysis. The whole kidneys were isolated immediately from the animals. The kidneys were washed with ice-cold physiological saline, and stored at −20°C for enzymatic analysis.

### 2.5. Renal Function Analysis

The serum urea and creatinine levels were measured using a commercial kit (Diasis Diagnostic Systems, Istanbul, Turkey).

### 2.6. Analysis of Oxidants and Antioxidants

Isolated kidney tissues were homogenized in a homogenizer using a buffer of 1.15% KCl to obtain a 1:10 (*w*/*v*) homogenate. According to methods of Placer et al. [25], the malondialdehyde (MDA) levels in the kidney homogenate were measured using thiobarbituric acid reactive substances (TBARS) [26]. The formed MDA created a pink complex with thiobarbituric acid (TBA) and the absorbance was read at 532 nm. The MDA levels of kidney tissue are expressed as nmol/g tissue. Kidney catalase (CAT) activity was measured according to the method of Aebi et al. [27]. The decomposition rate of H_2_O_2_ by CAT was spectrophotometrically measured as the amount of change in H_2_O_2_ light absorption at the 240 nm wavelength, and is expressed as katal/g of protein. Protein concentration was measured according to the method of Lowry et al. [28]. Superoxide dismutase (SOD) activity was measured according to the method of Sun et al. [29]. The SOD enzyme activity measurement is based on the nitroblue tetrazolium (NBT) degradation by the superoxide radical, which was produced with the xanthine-xanthine oxidase system and formazan; its creation was measured at 560 nm and is expressed as U/g protein. Decreased renal tissue glutathione (GSH) levels were measured by colour change at 412 nm according to the method of Sedlak and Lindsay, [30] and it is expressed as nmol/g of kidney tissue. The glutathione peroxidase (GPx) activity in kidney tissue was measured according to the method of Lawrence and Burk [31] and is expressed as U/g of protein.

### 2.7. Tissue Preparation for Autophagy, Inflammation and Apoptosis Markers

After cutting renal tissue samples, weight was checked and PBS (pH 7.2–7.4) was added. Tissue was homogenized by tissue lyser and centrifuged for 20 min at the speed of 1200× *g* to remove supernatant. Supernatant was used in inflammation, apoptosis, and autophagy analysis.

### 2.8. Assay of Inflammation

Cytokine production in the kidney tissue was determined by enzyme-linked immunosorbent assay (ELISA) using commercial kits according to manufacturer’s instructions. Kidney tumour necrosis factor-alpha (TNF-α) (sensitivity: 5.127 ng/L, assay range: 8–1000 ng/L) and interleukin-1β (IL-1β) levels were measured using a rat ELISA kit (sensitivity: 20.118 pg/L, assay range: 25–8000 pg/L) from Sunred Biological Technology (Shangai, China). Interleukin-33 (IL-33) level was measured using a rat ELISA kit (sensitivity: 20.118 pg/L; the minimum detectable dose of IL33 is typically less than 3.0 pg/mL) from Uscn Life Science Inc. (Houston, TX, USA). The plates were read at 450 nm using the ELISA microplate reader (Bio-Tek, Winooski, VT, USA).

### 2.9. Assay of Apoptosis

Cysteine aspartate specific protease-3 (Caspase-3) activity was determined using a kit (sensitivity: 0.045 ng/mL, assay range: 0.05–12 ng/mL) purchased from Sunred biological technology (Shangai, China) and was performed according to manufacturer’s instructions.

### 2.10. Assay of Autophagy

Light chain 3B (LC3B) levels were measured using rat ELISA kits (sensitivity: 0.27 ng/mL, assay range: 0.3–90 ng/mL) from Sunred Biological Technology (Shangai, China). The plates were read at 450 nm using the ELISA microplate reader.

### 2.11. Statistical Analysis

The data were performed with one-way ANOVA using the SPSS statistical program (Version 12.0; SPSS, Chicago, IL, USA). Duncan’s multiple range test (DMRT) was used to compare the studied parameters between the groups. The data are presented as the mean ± standard error of means (SEM). Differences were considered significant when the *p* < 0.05.

## 3. Results

### 3.1. Serum Biochemical Analysis

Serum urea and creatinine levels were significantly increased in PC-treated rats, compared to the control (273%) and CR-treated rats (307%) (*p* < 0.05). Levels of serum urea and creatinine in PC+CR-25 and PC+CR-50 were significantly decreased (34% and 51% for urea, 43% and 54% for creatinine) compared to PC-treated in rats (*p* < 0.05).

### 3.2. MDA Levels

There was a significant increase in the kidney MDA level in the PC-treated group as compared to control (90%) and CR-treated groups (101%) (*p* < 0.05). The kidney MDA level in the PC+CR-25 and PC+CR-50 groups were significantly decreased (26% and 36% respectively) compared to PC-treated rats (*p* < 0.05).

### 3.3. Antioxidant Enzymes

The SOD, CAT and GPx activities were not significantly increased in the CR-treated groups compared to the control group (*p* < 0.05). Activities of these enzymes were significantly decreased in the PC-treated groups compared to the control and CR-treated groups (*p* < 0.05). In the PC+CR-25 and PC+CR-50 groups, the SOD, CAT and GPx enzyme activities were significantly increased compared to the PC-treated group (*p* < 0.05).

There was no significant difference between renal GSH level of control and CR-treated groups (*p* < 0.05). The GSH level was significantly depleted in PC-treated group compared to control group. However, the GSH levels of the PC+CR-25 and PC+CR-50-treated groups were significantly increased as compared to the PC-treated group (*p* < 0.05). The results are shown in Table 1.

### 3.4. Inflammatory Markers

**Figure 1:**
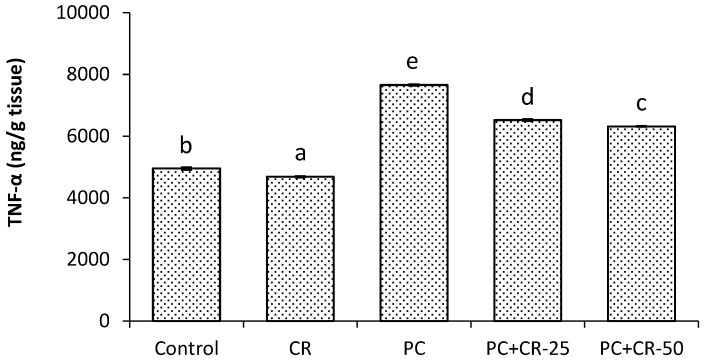
Effect of Chrysin (CR) and Paracetamol (PC) on tumour necrosis factor-alpha (TNF-α) in rat kidney tissues. Different letters (a, b, c, d and e) indicate statistically significant differences (*p* < 0.05) between groups.

There were minor differences in the kidney TNF-α level between control and CR-treated groups (*p* < 0.05). PC intoxication significantly increased the TNF-α level compared to the control group. However, TNF-α levels in the PC+CR-25-treated and PC+CR-50-treated groups were significantly decreased (15% and 18% respectively) compared to the PC-treated rats (*p* < 0.05). The results are shown in Figure 1.

**Figure 2:**
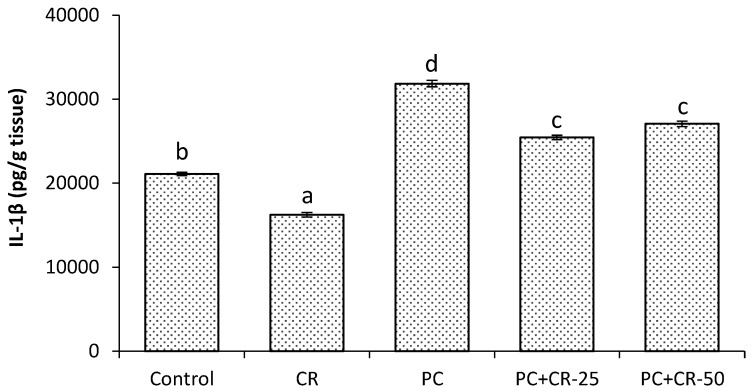
Effect of Chrysin (CR) and Paracetamol (PC) on interleukin-one beta (IL-1β) in rat and kidney tissues. Different letters (a, b, c, d and e) indicate statistically significant differences (*p* < 0.05) between groups.

There were significant differences in the kidney IL-1β level between control and CR-treated groups (*p* < 0.05). There was a significant increase in the kidney IL-1β level in the PC-treated group as compared to control and CR-treated groups (*p* < 0.05). The kidney IL-1β level in the PC+CR-25-treated and PC+CR-50-treated groups were significantly decreased (21% and 16% respectively) compared to PC-treated rats (*p* < 0.05, Figure 2).

**Figure 3:**
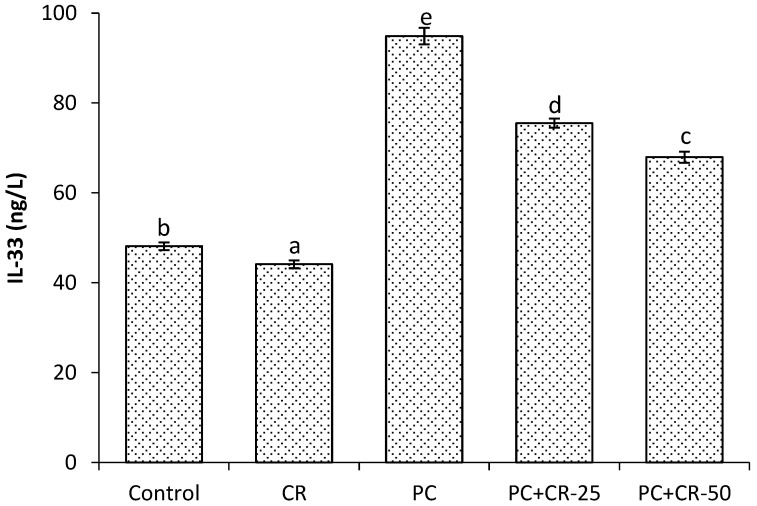
Effect of Chrysin (CR) and Paracetamol (PC) on interleukin-33 (IL-33) in rat and kidney tissues. Different letters (a, b, c, d and e) indicate statistically significant differences (*p* < 0.05) between groups.

There was a relative difference in the kidney IL-33 level between control and CR-treated groups (*p* < 0.05). There was a significant increase in the kidney IL-33 level in PC-treated group as compared to control and CR-treated groups (*p* < 0.05). The kidney IL-33 level in the PC+CR-25-treated and PC+CR-50-treated groups were significantly decreased (21% and 29% respectively) compared to PC-treated rats (*p* < 0.05, Figure 3).

### 3.5. Apoptotic Marker

**Figure 4:**
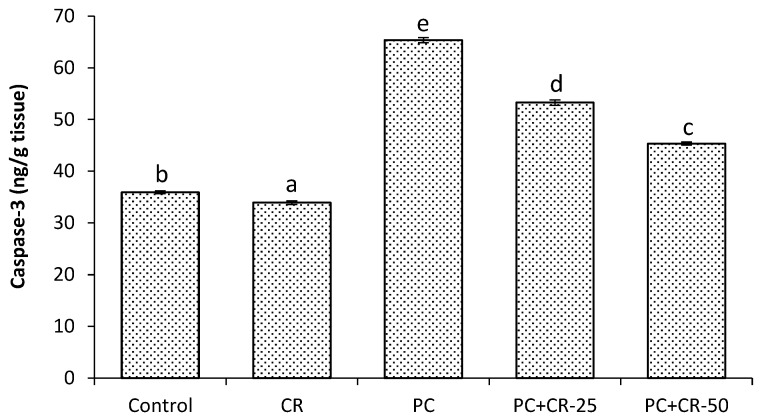
Effect of Chrysin (CR) and Paracetamol (PC) on cysteine aspartate-specific protease-3 (caspase 3) activity in rat and kidney tissues. Different letters (a, b, c, d and e) indicate statistically significant differences (*p* < 0.05) between groups.

Apoptosis is determined by assessing caspase-3 activity. The PC-treated group had significantly higher levels of caspase-3 compared to control and CR-treated groups (*p* < 0.05). On the other hand, PC+CR-25- and PC+CR-50-treated groups exhibited an anti-apoptotic effect of treatment by significantly decreased (19% and 31% respectively) caspase-3 activity compared to the PC-treated group (*p* < 0.05). As shown in Figure 4, the PC+CR-50-treated group had greater decreases in caspase-3 activity compared to the PC+CR-25-treated group (*p* < 0.05, Figure 4).

### 3.6. Autophagic Marker

**Figure 5:**
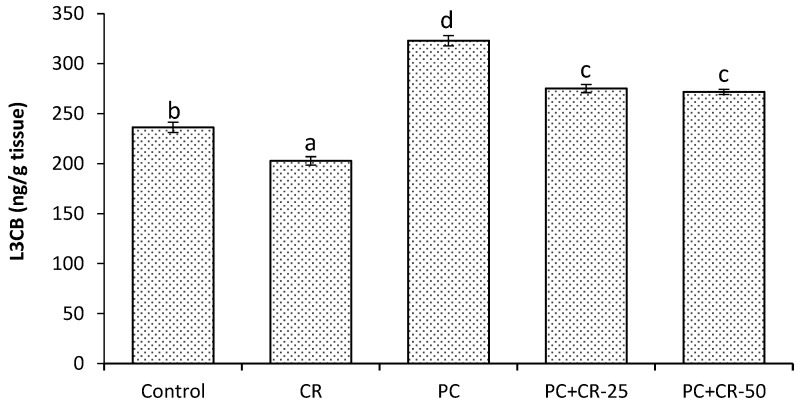
Effect of Chrysin (CR) and Paracetamol (PC) on light chain 3B (LC3B) level in rat and kidney tissues. Different letters (a, b, c, d and e) indicate statistically significant differences (*p* < 0.05) between groups.

There was a significant difference in the kidney LC3B level between control and CR-treated groups (*p* < 0.05). There was a significant increase in the kidney LC3B level in the PC-treated group as compared to control and CR-treated groups. In the PC+CR-25 and PC+CR-50 groups, the kidney LC3B level was significantly decreased (15% and 17% respectively) at almost the same amount compared to the PC-treated group (*p* < 0.05). The results are summarized in the Figure 5.

## 4. Discussion

The kidneys are important organs that play a role in all humans and animals. The general functions of the kidneys are to regulate blood pressure, acid-base balance, electrolyte balance and extracellular fluid volume. They also eliminate substances from the body including metabolic products, various toxins and other foreign substances such as drugs, pesticides and food additives [32,33]. It has been reported that high doses of therapeutic agents such as aminoglycoside, antibiotics, chemotherapeutic agents and non-steroidal analgesic drugs induce kidney toxicity [34]. PC is a non-steroidal analgesic and antipyretic drug that has widespread use. The drug is effective and safe in therapeutic doses, but acute or cumulative doses of the drug may induce renal tubular necrosis in both humans and laboratory animals [35]. Increases in serum urea and creatinine levels are an important marker of renal toxicity. Kidney disease and function disorders may occur as a result of serum urea accumulation exceeding its clearance rate. Similarly, increases in plasma creatinine levels are considered a nephron function disorder [36]. An overdose of PC causes many metabolic disturbances including an increase in serum urea and creatinine [32]. In the present study, serum urea and creatinine levels in the groups treated with PC+CR-25 and PC+CR-50 were significantly reduced compared with the PC-treated group. CR pre-treatment attenuated renal damage by reducing serum toxicity markers such as urea and creatinine against the PC-induced nephrotoxicity.

MDA is known as a secondary product of lipid peroxidation and is used as a marker of tissue damage resulting from many chain reactions [37,38]. Some studies have reported that renal oxidative stress is induced by increases in lipid peroxidation products (e.g., MDA) and decreases in antioxidant defensive enzyme activities [39,40]. The previous study shows that PC-induced rats had significant lipid hydroperoxides in kidney tissue [41]. Increased MDA levels in kidney and other tissues have long been known to cause functional degradation; therefore, the degradation of vital tissue leading to complications may be indirectly due to increased oxidative stress [21]. In our study, the administration of PC (500 mg/kg) caused significant elevations in kidney MDA levels compared to the control group. Alternatively, the MDA levels in the PC+CR-25 and PC+CR-50 groups were significantly lower compared to the PC-treated group. Our results showed that CR reduced MDA levels in the kidney tissue. CR provides a satisfactory protection against PC-induced lipid peroxidation.

Enzymatic antioxidants play an important role by protecting the cells exposed to oxidative damage. SOD is an important enzyme that converts the superoxide radical (O_2_·^−^) to molecular oxygen (O_2_) and hydrogen peroxide (H_2_O_2_) [42,43,44]. CAT is an enzyme that converts H_2_O_2_ to O_2_ and water, and is generally located in the intracellular peroxisomes [45,46]. GPx is an enzyme that converts H_2_O_2_ and lipid hydroperoxides to water. The activities of the SOD, CAT and GPx enzymes were significantly reduced in the PC-induced kidney toxicity group. In this study, PC significantly reduced the antioxidant activities of SOD, CAT, and GPx in the renal tissue in comparison with the control group. Pre-treatment with 25 mg/kg CR and 50 mg/kg CR significantly attenuated the PC-induced decrease in antioxidant enzyme activities observed in rat kidney tissue. CR treatment also significantly reduced PC-induced oxidative stress levels. The mechanism of this effect is considered to be due to the capability of the hydroxyl groups located on the fifth and eighth positions in the CR molecule to directly eliminate free radicals [19]. Additionally, it has been determined that CR indirectly suppresses oxidative stress by regulating antioxidant enzyme activities [47]. Similarly, many studies have reported that CR treatment protects tissues against oxidative stress and induces an increase in antioxidant enzyme activities [21,48].

GSH is a non-enzymatic tripeptide protecting the tissues and organs against the adverse effects of ROS. It plays a role in eliminating free radical species such as H_2_O_2_, superoxide radicals and membrane protein thiols [49,50]. It is also known as a substrate of the GPx enzyme [51]. In therapeutic doses, PC directly establishes a conjugated bond with glucuronic acid and sulphate, creating non-toxic conjugated metabolites that are discarded by the kidneys. On the other hand, high doses of PC are metabolized to NAPQI (likely via reaction with GSH in the cytochrome P450 system), which is a reactive intermediate product. When GSH stores are depleted, the remaining NAPQI binds to macromolecules in cellular proteins. This leads to tissue necrosis and stimulates programmed necrosis or apoptosis, as well as deteriorating homeostasis, and ultimately induces mitochondrial function disorders [3,33,52]. The 25 mg/kg CR and 50 mg/kg CR treatments significantly attenuated the decreases in cellular GSH amounts that were seen in the PC-induced rat kidney tissue. Khan et al. [53] also reported that CR applications increased GSH stores.

In this study, we determined that the PC toxic dose induced an inflammatory reaction by increasing the TNF-α levels. However, CR therapy provided an ameliorative effect against kidney toxicity induced by high doses of PC. These results suggest that CR has anti-inflammatory effects by reducing pro-inflammatory cytokine expression. Furthermore, CR has the capability of inhibiting pro-inflammatory cytokines by reducing the inflammation response in macrophages and monocytes [54]. IL-1β is produced by macrophages activated as proteins and is also known as catabolin. This cytokine proliferation is known as an important mediator of the inflammatory response and various cellular functions such as differentiation and apoptosis [55]. IL-33 is a cytokine belonging to the IL-1 family. It is excreted throughout the entire body and recently discovered as a signal receptor [55,56]. In this study, IL-1β and IL-33 levels were significantly increased in the group given PC in comparison with the control group. On the other hand, CR significantly decreased levels of both IL-1β and IL-33. These data demonstrate that CR exerts anti-inflammatory effects by decreasing levels of various pro-inflammatory cytokines.

Some studies have determined that high doses of PC lead to both apoptosis in several tissues and significantly increased caspase-3 levels [57]. In this study, caspase-3 activity significantly increased in the group treated with PC and treatment with CR significantly decreased caspase-3 activity. It has been reported that CR administration reduces the parenchyma in renal tissue along with apoptosis in the glomerular and tubular regions [58].

A similar study demonstrated that PC in high doses induces autophagia activation in rat tissue [59]. LC3B is a membrane structural protein and a frequently used biomarker for autophagia [60]. Lazova et al. [61] demonstrated that LC3B expression increased in various malignancies and they reported that autophagia played an important role in the cancer process. In our present study, we observed that LC3B levels were significantly increased in the PC-treated group compared to the controls. However, Figure 5 shows that LC3B levels were reduced in the groups given CR treatments. In a similar study, it was reported that CR protected glioblastoma cells against treatment with chronic temozolomide and inhibited temozolomide-induced autophagia [62].

## 5. Conclusions

In conclusion, our results confirmed the anti-oxidative, anti-inflammatory, anti-apoptotic, and anti-autophagic effects of CR on PC-induced kidney damage in rats. Therefore, supportive treatment of CR demonstrated beneficial effect against PC-induced kidney damage.

## Figures and Tables

**Table 1:** Effect of CR and PC on serum markers and MDA, GSH and antioxidant enzymes in rat kidney tissue.

Parameter	Control	CR	PC	PC+CR-25	PC+CR-50
**Urea** (mg/dL)	6.26 ± 0.09 ^a^	5.74 ± 0.12 ^a^	23.37 ± 0.75 ^d^	15.53 ± 0.3 ^c^	11.50 ± 0.35 ^b^
**Creatinine** (mg/dL)	0.72 ± 0.01 ^a^	0.67 ± 0.01 ^a^	2.74 ± 0.04 ^d^	1.57 ± 0.02 ^c^	1.27 ± 0.04 ^b^
**MDA** (nmol/g tissue)	63.26 ± 0.98 ^a^	59.81 ± 0.57 ^a^	120.39 ± 3.3 ^d^	89.35 ± 1.14 ^c^	76.94 ± 1.04 ^b^
**SOD** (U/g protein)	30.32 ± 0.41 ^c^	31.40 ± 0.25 ^c^	22.30 ± 0.58 ^a^	25.47 ± 0.29 ^b^	24.78 ± 1.53 ^b^
**GPx** (U/g protein)	36.26 ± 0.40 ^d^	37.64 ± 0.38 ^d^	24.80 ± 0.54 ^a^	30.32 ± 0.59 ^b^	33.68 ± 0.55 ^c^
**CAT** (katal/g protein)	65.95 ± 1.40 ^d^	68.68 ± 0.87 ^d^	45.36 ± 0.78 ^a^	51.18 ± 0.66 ^b^	56.22 ± 0.92 ^c^
**GSH** (nmol/g tissue)	4.37 ± 0.05 ^d^	4.58 ± 0.04 ^d^	1.70 ± 0.03 ^a^	2.28 ± 0.04 ^b^	2.98 ± 0.06 ^c^

CR, chrysin; PC, paracetamol; MDA, malondialdehyde; SOD, superoxide dismutase; CAT, catalase; GPx, glutathione peroxidase; GSH, glutathione. The values are expressed as Mean ± SEM of seven rats in each group. Different superscripts (a, b, c and d) in the same row indicate statistically significant difference (*p* < 0.05) between groups.
